# Advanced Hodgkin lymphoma in the East of England: a 10-year comparative analysis of outcomes for real-world patients treated with ABVD or escalated-BEACOPP, aged less than 60 years, compared with 5-year extended follow-up from the RATHL trial

**DOI:** 10.1007/s00277-021-04460-9

**Published:** 2021-02-27

**Authors:** James Russell, Angela Collins, Alexis Fowler, Mamatha Karanth, Chandan Saha, Suzanne Docherty, Joseph Padayatty, Kyaw Maw, Isabel Lentell, Lisa Cooke, Andrew Hodson, Nimish Shah, Shalal Sadullah, Nicholas Grigoropoulos, Wendi Qian, Amy A. Kirkwood, Benjamin J. Uttenthal, Peter Johnson, George A. Follows

**Affiliations:** 1 Department of Haematology, Addenbrooke’s Hospital, Cambridge University Hospitals NHS Foundation Trust, Hills Road, Cambridge, CB2 0QQ UK; 2 Department of Haematology, Norfolk and Norwich University Hospital, Norfolk and Norwich University Hospitals NHS Foundation Trust, Colney Lane, Norwich, NR4 7UY UK; 3 Department of Haematology, Peterborough City Hospital, Peterborough and Stamford Hospitals NHS Foundation Trust, Edith Cavell Campus, Bretton Gate, Peterborough, PE3 9G UK; 4 Department of Haematology, West Suffolk Hospital, West Suffolk NHS Trust, Hardwick Lane, Bury St Edmunds, IP33 2QZ UK; 5 Department of Haematology, James Paget University Hospital, James Paget University Hospitals NHS Foundation Trust, Lowestoft Road, Gorleston-on-Sea, Great Yarmouth, NR31 6LA UK; 6 Department of Haematology, The Queen Elizabeth Hospital, King’s Lynn NHS Foundation Trust, Gayton Rd, King’s Lynn, PE30 4ET UK; 7 Department of Haematology, Ipswich Hospital, Ipswich Hospital NHS Trust, Heath Rd, Ipswich, IP4 5PD UK; 8 Cancer Research UK and University College London Cancer Trials Centre, University College London, 90 Tottenham Court Road, London, W1T 4TJ UK; 9 Cancer Research UK Centre, University of Southampton, University Road, Southampton, SO17 1BJ UK

**Keywords:** Hodgkin lymphoma, Advanced stage, Response-adapted therapy, Real-world data, ABVD, Escalated BEACOPP

## Abstract

Treatment with ABVD (doxorubicin, bleomycin, vinblastine, and dacarbazine) or escalated(e)-BEACOPP (bleomycin, etoposide, doxorubicin, cyclophosphamide, vincristine, procarbazine, and prednisolone) remains the international standard of care for advanced-stage classical Hodgkin lymphoma (HL). We performed a retrospective, multicentre analysis of 221 non-trial (“real-world”) patients, aged 16–59 years, diagnosed with advanced-stage HL in the Anglia Cancer Network between 2004 and 2014, treated with ABVD or eBEACOPP, and compared outcomes with 1088 patients in the Response-Adjusted Therapy for Advanced Hodgkin Lymphoma (RATHL) trial, aged 18–59 years, with median follow-up of 87.0 and 69.5 months, respectively. Real-world ABVD patients (*n*=177) had highly similar 5-year progression-free survival (PFS) and overall survival (OS) compared with RATHL (PFS 79.2% vs 81.4%; OS 92.9% vs 95.2%), despite interim positron-emission tomography-computed tomography (PET/CT)-guided dose-escalation being predominantly restricted to trial patients. Real-world eBEACOPP patients (*n*=44) had superior PFS (95.5%) compared with real-world ABVD (HR 0.20, *p*=0.027) and RATHL (HR 0.21, *p*=0.015), and superior OS for higher-risk (international prognostic score ≥3 [IPS 3+]) patients compared with real-world IPS 3+ ABVD (100% vs 84.5%, *p*=0.045), but not IPS 3+ RATHL patients. Our data support a PFS, but not OS, advantage for patients with advanced-stage HL treated with eBEACOPP compared with ABVD and suggest higher-risk patients may benefit disproportionately from more intensive therapy. However, increased access to effective salvage therapies might minimise any OS benefit from reduced relapse rates after frontline therapy.

## Introduction

Treatment with ABVD (doxorubicin, bleomycin, vinblastine, and dacarbazine) or escalated(e)-BEACOPP (bleomycin, etoposide, doxorubicin, cyclophosphamide, vincristine, procarbazine, and prednisolone) remains the international standard of care for the management of adult patients with advanced-stage classical Hodgkin lymphoma (HL). In 2009, 10-year follow-up data from the German Hodgkin Study Group (GHSG) HD9 trial showed a significant improvement in both disease control (time-to-treatment-failure) and overall survival (OS) for patients treated upfront with standard-dose BEACOPP or eBEACOPP, compared with alternating cycles of COPP (cyclophosphamide, vincristine, procarbazine, and prednisone) and ABVD [1]. Subsequently, eBEACOPP was introduced as a treatment option at six cancer centres in the Anglia Cancer Network (ACN) for certain patients for whom it was considered clinically appropriate, whilst two other ACN centres continued to offer ABVD for all patients. From 2008 to 2012, four of the ACN centres also recruited patients onto the Response-Adjusted Therapy for Advanced Hodgkin Lymphoma (RATHL) trial. In RATHL, all patients received two cycles of ABVD followed by an interim positron-emission tomography-computed tomography (PET/CT) scan (iPET2) to guide further treatment intensity. Patients with a negative iPET2 scan (Deauville score 1–3) were randomised 1:1 to receive a further four cycles of ABVD or AVD (without bleomycin). Patients with a positive iPET2 scan (Deauville score 4–5) had their treatment intensified and received either a further three cycles of eBEACOPP or four cycles of BEACOPP-14 (standard-dose BEACOPP delivered every 14 days), without randomisation. Radiotherapy (RT) was not mandated in the trial protocol but could be offered at the discretion of the treating clinician. The 3-year follow-up results of RATHL have been published [2].

In our retrospective study, we looked specifically at young patients, aged 16–59 years, with advanced-stage HL who were treated with first-line ABVD or eBEACOPP in the ACN over a 10-year period (2004–2014), which included the 5 years immediately preceding and following the introduction of eBEACOPP at six ACN centres in 2009. We compared our outcomes against an updated 5-year analysis of outcomes for RATHL patients, aged 18–59 years. This allowed us to compare progression-free survival (PFS) and OS for patients treated upfront with ABVD or eBEACOPP in a non-trial (so-called real-world), multicentre setting, against patients treated in a large, contemporary ABVD-based prospective trial that incorporated interim-PET/CT-guided dose-escalation for patients with an inferior response after two cycles of standard ABVD induction therapy.

## Methods

### Data collection

We collected data retrospectively from eight cancer centres in the Anglia Cancer Network (ACN), including Cambridge University Hospital and Nuffield Hospital, Cambridge; Norfolk and Norwich University Hospital, Norwich; Ipswich Hospital, Ipswich; James Paget University Hospital, Great Yarmouth; Hinchingbrooke Hospital, Hinchingbrooke; Peterborough City Hospital, Peterborough; Queen Elizabeth Hospital, King’s Lynn; and West Suffolk Hospital, Bury Saint Edmunds. Eligible patients were identified by review of electronic and paper hospital records, local cancer registry data, and the NHS database. Study inclusion criteria included all young (aged 16–59 years), treatment-naïve patients diagnosed with advanced-stage HL (stage IIB–IV, or IIA with bulk disease defined as >0.33 transthoracic diameter or >10cm) in the ACN between July 1, 2004, and June 31, 2014, who were treated outside of a clinical trial (so-called real-world) with either upfront ABVD or eBEACOPP. To provide comparable durations of follow-up, real-world ABVD and eBEACOPP patients were censored in April 2016 and January 2019, respectively, and RATHL trial patients were censored in June 2019.

### Statistical analysis

Statistical analysis was conducted using Stata version 16.1 software (StataCorp, TX, USA). Baseline variables of interest were calculated using standard summary statistics and included age at diagnosis, sex, biopsy date, Ann Arbor stage, international prognostic score (IPS), treatment regimen, use of radiotherapy, date of relapse and/or death. Pearson’s chi-squared test for categorical variables was applied for the comparison between groups. Survival analysis was calculated from the date of diagnostic biopsy to the date of first progression or death from all causes. Surviving patients without progression were censored at the date they were last known to be alive. Survival estimates with 95% confidence intervals were calculated using the Kaplan-Meier method and compared using the log-rank test. Hazard ratios (HR) with 95% confidence intervals were calculated using a Cox regression model. A *p*-value of <0.05 was considered significant. Median follow-up duration was calculated using the reverse Kaplan-Meier method, and was censored at death from all causes. Statistical analysis of the 5-year follow-up data from the RATHL trial for patients aged 18–59 years, and subsequent comparative analysis with our real-world ACN cohort, was performed by the RATHL trial team.

## Results

**Fig. 1:**
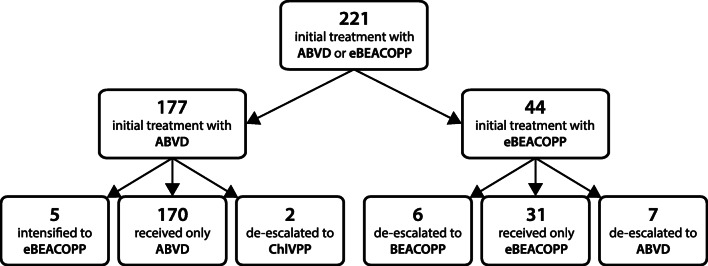
Flowchart of the treatment pathway for 221 young advanced-stage classical Hodgkin lymphoma patients diagnosed in the Anglia Cancer Network, from 2004 to 2014. ABVD, doxorubicin, vinblastine, bleomycin, and dacarbazine; BEACOPP, bleomycin, etoposide, doxorubicin, cyclophosphamide, vincristine, procarbazine, and prednisolone; ChlVPP, chlorambucil, vinblastine, procarbazine, and prednisolone; eBEACOPP, escalated-BEACOPP

We identified 250 patients, aged 16–59 years, diagnosed with advanced-stage HL in the ACN from July 1, 2004, to June 31, 2014. This number is in keeping with the expected incidence for the ACN population of 2.64 million [3]. Of these 250 patients, 29 were excluded, including 25 who were treated in the RATHL trial, three who were treated with regimens other than ABVD or eBEACOPP, and one who died before commencing therapy. There were 221 patients eligible for study inclusion, with a median follow-up of 87 months. A flowchart of the treatment pathway for the whole cohort is shown in Fig. 1. Real-world patient outcomes were compared with 5-year outcomes for patients treated in the RATHL trial, aged 18–59 years (*n*=1088), with a median follow-up of 69.5 months.

Comparison of the baseline characteristics of real-world and RATHL patients showed highly similar age and sex distribution, but the real-world cohort had significantly fewer stage II patients and significantly more stage IV (*p* < 0.001) and higher risk (IPS 3+) patients (*p* = <0.001), compared with the RATHL trial (Table 1). In the real-world cohort, there was a patient–clinician bias to treat higher-risk patients with more-intensive induction therapy, with 75% of eBEACOPP patients being IPS 3+ compared with only 39% of ABVD patients (*p*<0.001)

**Table 1:** Baseline characteristics of 221 young advanced-stage Hodgkin lymphoma patients treated in the real-world (RW) setting compared with 1088 patients treated in the RATHL trial. *ABVD*, doxorubicin, vinblastine, bleomycin, and dacarbazine; *eBEACOPP*, escalated-bleomycin, etoposide, doxorubicin, cyclophosphamide, vincristine, procarbazine, and prednisolone; *RATHL*, response-adapted therapy for advanced Hodgkin lymphoma

Baseline characteristics	Real-world, *N*=221	RATHL, *N*=1088
Median age at diagnosis—y (range)	35 (16–59)	31 (18–59)
Male sex—*n* (%)	121 (54.8)	585 (53.8)
Median follow-up—mo. (95% CI)		
Whole cohort	87.0 (83.0–94.0)	69.5 (67.2–69.5)
ABVD	87.0 (83.0–94.3)	–
eBEACOPP	88.0 (79.3–102.1)	–
Ann Arbor stage—*n* (%)		
II with adverse features	62 (28.1)	471 (43.3)
III	68 (30.8)	312 (28.7)
IV	91 (41.2)	305 (28.0)
International Prognostic Score—*n* (%)		
0–2	117 (52.9)	728 (67.0)
3–6	102 (46.2)	359 (33.0)
Unknown	2 (0.9)	1 (0.0)
Initial treatment—*n* (%)		
ABVD	177 (80.1)	1088 (100.0)
eBEACOPP	44 (19.9)	0 (0.0)

**Fig. 2:**
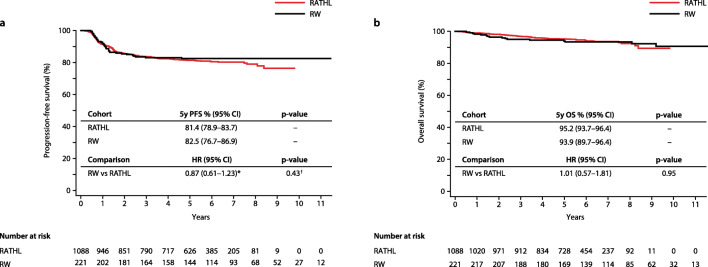
Kaplan-Meier curves of survival estimates for the whole cohort of real-world (RW) patients compared with RATHL trial patients. **a** Progression-free survival (PFS). **b** Overall survival (OS). HR, hazard ratio; RATHL, response-adapted therapy for advanced Hodgkin lymphoma; †log rank *p*-value; *fails the assumption of proportional hazards

The 5-year PFS and OS estimates for the whole cohort of real-world patients (*n*=221) were 82.5% and 93.9%, respectively. These results were highly similar for RATHL patients aged 18–59 years (*n*=1088); PFS 81.4%; HR 1.05, *p* = 0.79; OS 95.2%, *p*=0.71; Fig. 2 Of the 177 real-world ABVD patients, the majority (*n*=130; 73.4%) completed six cycles of ABVD, and 41 (23.2%) completed eight cycles. Interim-PET/CT scans were not routinely performed in the real-world setting. Five patients (2.8%) had their treatment intensified to eBEACOPP after two, three, or four cycles of ABVD, and two patients (1.1%) were de-escalated to ChlVPP (chlorambucil, vinblastine, procarbazine, and prednisolone) after three cycles of ABVD. Bleomycin was stopped after two or three cycles in 15 patients (8.5%) because of pulmonary toxicity. Twenty patients of the ABVD cohort (11%) received consolidative radiotherapy (RT) after completing frontline chemotherapy, including 16/20 who were treated exclusively with ABVD therapy (15/16 received RT to areas of residual bulk and 1/16 for progressive disease), 3/20 who had their treatment intensified to eBEACOPP (3/3 received IFRT to areas of residual bulk), and 1/20 who had their treatment de-escalated to ChlVPP). By comparison, 154 (14%) RATHL patients had their treatment intensified to eBEACOPP or BEACOPP-14 after two cycles of ABVD as per trial protocol, and 73 (6.7%) patients received consolidative RT [2].

**Fig. 3:**
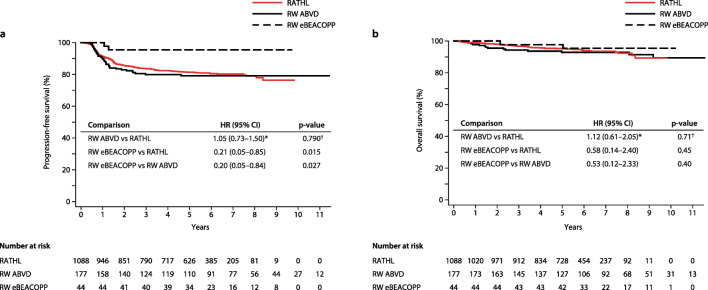
Kaplan-Meier curves of survival estimates for real-world (RW) patients, grouped by treatment regimen, compared with RATHL trial patients. **a** Progression-free survival (PFS). **b** Overall survival (OS). ABVD, doxorubicin, vinblastine, bleomycin, and dacarbazine; eBEACOPP, escalated-bleomycin, etoposide, doxorubicin, cyclophosphamide, vincristine, procarbazine, and prednisolone; HR, hazard ratio; RATHL, response-adapted therapy for advanced Hodgkin lymphoma; †log rank *p*-value; *fails the assumption of proportional hazards

Of the 44 real-world eBEACOPP patients, 31 (70.5%) were treated exclusively with eBEACOPP, of which the majority (*n*=25; 80.6%) completed six cycles, four (12.9%) completed five cycles, one (3.2%) completed seven cycles, and one (3.2%) completed eight cycles. Six patients (13.6%) were de-escalated to standard-dose BEACOPP after either two (*n*=3, 6.8%), three (*n*=1, 2.3%), or four (*n*=2, 4.5%) cycles of eBEACOPP. Seven patients (15.9%) were de-escalated to ABVD after one (*n*=1, 2.3%), two (*n*=4, 9.1%), three (*n*=1, 2.3%), or four (*n*=1, 2.3%) cycles of eBEACOPP. De-escalation was primarily required because of toxicity; however, three patients requested de-escalation due to fertility concerns. Of the 44 eBEACOPP patients, 5 patients (11%) received consolidative RT to areas of residual nodal tissue > 2cm with residual metabolic uptake on PET/CT. No patients in this cohort had RT for primary refractory disease. Kaplan-Meier curves of PFS and OS for real-world patients, by treatment regimen, and RATHL trial patients are shown in Fig. 3.

The 5-year PFS and OS estimates for real-world ABVD patients (*n*=177) were 79.2% and 92.9% respectively. Survival outcomes were highly similar in RATHL patients, aged 18–59 years; PFS 81.4%, HR 1.05, *p*=0.79; OS 95.2%, *p*=0.71. Although the real-world eBEACOPP cohort comprised proportionately more higher-risk patients (75% were IPS 3+, compared with 39% of real-world ABVD patients and 33% of RATHL patients), it had a statistically significant PFS advantage compared with the real-world ABVD cohort (HR 0.20, *p*=0.027) and RATHL trial (HR 0.21, *p*=0.015; Fig. 3a). However, there was no statistically significant OS advantage (Fig. 3b).

**Fig. 4:**
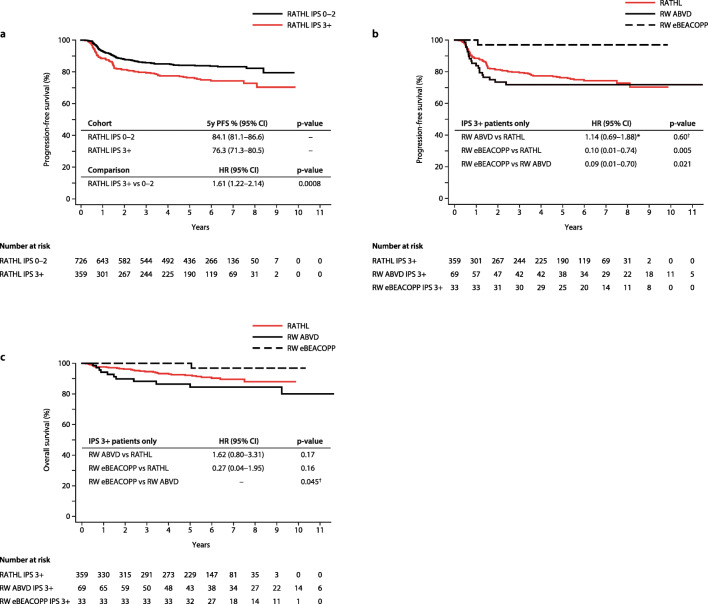
Kaplan-Meier curves of survival estimates for real-world (RW) and RATHL trial patients, grouped by treatment regimen and international prognostic score (IPS). **a** Progression-free survival (PFS) for RATHL trial patients, grouped by IPS. **b** PFS for IPS 3+ real-world patients, grouped by treatment regimen, compared with IPS 3+ RATHL trial patients. **c** Overall survival (OS) for IPS 3+ real-world patients, grouped by treatment regimen, compared with IPS 3+ RATHL trial patients. ABVD, doxorubicin, vinblastine, bleomycin, and dacarbazine; eBEACOPP, escalated-bleomycin, etoposide, doxorubicin, cyclophosphamide, vincristine, procarbazine, and prednisolone; HR, hazard ratio; RATHL, response-adapted therapy for advanced Hodgkin lymphoma; † log rank *p*-value; *fails the assumption of proportional hazards

With the RATHL trial, a higher IPS was associated with a poorer response to ABVD induction therapy, with 22.1% of IPS 3+ patients being iPET2-positive versus 12.9% of IPS 0–2 patients. With ongoing follow-up, IPS remains prognostic of 5-year PFS (HR 1.61, *p*=0.0008) despite a greater proportion of IPS 3+ patients receiving treatment intensification (Fig. 4a).

Real-world IPS 3+ eBEACOPP patients had significantly improved 5-year PFS and OS estimates compared with real-world IPS 3+ ABVD patients (PFS 97.0% vs 71.9%; HR 0.09, *p*=0.021, Fig. 4b; OS 100% vs 84.5%, *p*=0.0449, Fig. 4c). In addition, real-world IPS 3+ eBEACOPP patients retained a significant PFS benefit compared with IPS 3+ RATHL patients (97.0% vs 76.3%; HR 0.10, *p*=0.005; Fig. 4b) despite 22.1% of IPS 3+ RATHL patients receiving treatment intensification after two cycles of ABVD, as per protocol. Although a higher OS rate was observed for the real-world IPS 3+ eBEACOPP patients compared to the IPS 3+ RATHL patients (100% vs 91.4%; HR 0.27), this did not reach statistical significance (*p*=0.16) (Fig. 4c).

The 5-year survival estimates for real-world and RATHL trial patients are presented for all patient subgroups in Table 2.

**Table 2:** Summary of 5-year survival estimates for real-world (RW) and RATHL trial patients, grouped by treatment regimen, clinical stage, and international prognostic score (IPS). *ABVD*, doxorubicin, vinblastine, bleomycin, and dacarbazine; *eBEACOPP*, escalated-bleomycin, etoposide, doxorubicin, cyclophosphamide, vincristine, procarbazine, and prednisolone; *HR*, hazard ratio; *RATHL*, response-adapted therapy for advanced Hodgkin lymphoma; *fails the assumption of proportional hazards

Endpoint/group	RW ABVD 5-year rate % (*n*; 95% CI)		RW eBEACOPP 5-year rate % (*n*; 95% CI)	RATHL 5-year rate % (*n*; 95% CI)	RW ABVD vs RATHL HR (95% CI); *p*-value
Progression-free survival					
Whole cohort	79.2 (177; 72.3–84.5)		95.5 (44; 83.0–98.8)	81.4 (1088; 78.9–83.7)	1.05 (0.73–1.50); 0.79
IPS 0–2	84.7 (106; 76.2–90.3)		90.9 (11; 50.8–98.7)	84.1 (728; 81.1–86.6)	0.89 (0.52–1.50); 0.66
IPS 3+	71.9 (69; 59.5–81.1)		97.0 (33; 80.4–99.6)	76.3 (359; 71.3–80.5)	1.14* (0.69–1.88); 0.60
Stage IV	73.6 (59; 60.0–83.2)		96.9 (32; 79.8–99.6)	77.0 (305; 71.6–81.5)	1.04 (0.59–1.82); 0.90
Overall survival					
Whole cohort	92.9 (177; 87.8–95.9)		97.7 (44; 84.9–99.7)	95.2 (1088; 93.7–96.4)	1.12* (0.61–2.05); 0.71
IPS 0–2	98.1 (106; 92.5–99.5)		90.9 (11; 50.8–98.7)	96.7 (728; 95.0–97.9)	0.47 (0.14–1.59); 0.22
IPS 3+	84.5 (69; 72.9–91.4)		100 (33; N/A)	92.2 (359; 88.7–94.7)	1.62 (0.80–3.31); 0.18
Stage IV	92.6 (59; 81.3–97.2)		100 (32; N/A)	91.5 (305; 87.4–94.3)	0.82 (0.31–2.16); 0.69

### Fertility

Seventeen pre-menopausal women were treated with upfront eBEACOPP in our real-world cohort, of which 13 (76.5%) were younger than 30 years at diagnosis. Following completion of treatment, 12 (70.6%) regained menstrual periods (11 of whom were younger than 30 years at diagnosis), four (23.5%) were diagnosed with premature ovarian failure, and one (5.9%) died from complications related to allogeneic stem cell transplantation. At the time of data collection, five of these 17 women (29.4%) had had a total of seven pregnancies, which resulted in five live-births and two terminations of pregnancy.

### Use of stem cell transplantation

**Fig. 5:**
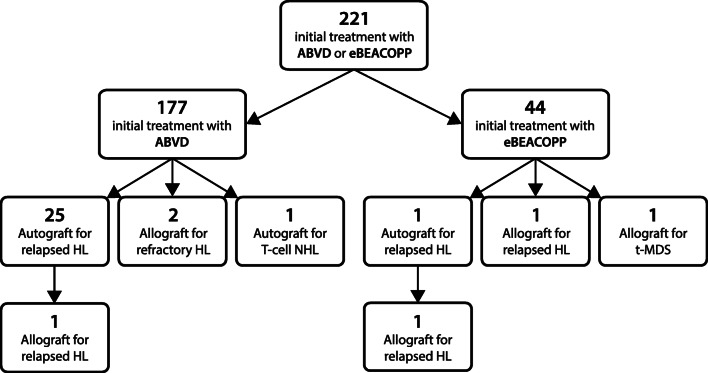
Flowchart of real-world patients treated with autologous (autograft) and/or allogeneic (allograft) stem cell transplantation for relapsed/refractory classical Hodgkin lymphoma (HL) or second malignancies, grouped by initial treatment regimen. ABVD, doxorubicin, vinblastine, bleomycin, and dacarbazine; eBEACOPP, escalated-bleomycin, etoposide, doxorubicin, cyclophosphamide, vincristine, procarbazine, and prednisolone; NHL, non-Hodgkin lymphoma; t-MDS, therapy-related myelodysplastic syndrome

Figure 5 shows a flowchart of real-world patients treated with stem cell transplantation (SCT) for relapsed/refractory HL or second malignancies, after frontline therapy with ABVD or eBEACOPP. There was a statistically significant difference in the rate of autologous SCT (autograft) between the real-world ABVD and eBEACOPP groups (*p*=0.0415). In the real-world ABVD cohort, 26 patients (14.7%) received an autograft; in 25 patients, this was after salvage therapy for relapsed HL, and in one patient as treatment of T-cell lymphoma. Three real-world ABVD patients (1.7%) had an allogeneic SCT (allograft) for relapsed HL, including one patient who had previously had an autograft.

In the real-world eBEACOPP group, one patient (2.3%) had an autograft after salvage therapy for relapsed HL, followed by an allograft after a second relapse. A further two eBEACOPP patients had an allograft, both without previous autograft, including one patient with relapsed HL who did not mobilise sufficient stem cells for an autograft after salvage chemotherapy and who received an allograft after a second relapse, and one patient with therapy-related myelodysplastic syndrome (t-MDS), comprising a total of three eBEACOPP patients (6.8%) who received an allograft compared with 1.7% in the ABVD group.

## Discussion

ABVD and eBEACOPP remain the most widely used first-line treatments of advanced-stage HL, and are recommended treatment options in both the current UK and European Society for Medical Oncology (ESMO) guidelines [4, 5]. It is broadly accepted that eBEACOPP offers more effective anti-Hodgkin therapy, but at the cost of additional toxicity. As such, it remains a case-by-case decision as to which regimen is more appropriate for a given patient.

In this retrospective study, we compared the outcomes of patients treated with upfront ABVD or eBEACOPP in a multicentre real-world setting, against patients treated in the RATHL trial. A strength of this study is the multi-hospital real-world dataset that has been rigorously checked to include all age-appropriate patients treated for Hodgkin lymphoma in the cancer network over a 10-year period. However, there are inherent limitations with real-world retrospective case studies which include treatment selection bias and particularly with our study, the relatively small number of patients and events in the eBEACOPP treatment cohort which inevitably introduces more uncertainty in the subsequent comparative statistical analysis. The over-representation of stage II patients in RATHL compared with other large prospective Hodgkin lymphoma trials has been the subject of previous discussion [6], but despite this limitation, it is the largest contemporary multicentre trial evaluating ABVD in the modern era. A similar but smaller trial was run by the US SWOG group (S0816) where iPET-positive patients were also intensified to eBEACOPP. In contrast with RATHL, however, only stage III/IV patients were included and dose-intensified patients received 6 rather than 4 cycles of eBEACOPP. The overall 5-year PFS for S0816 was 74% which appears inferior to RATHL, possibly reflecting the inclusion criteria for the trial. Both RATHL and SWOG S0816 reflect the modern practice of using minimal consolidative RT and we felt RATHL was an appropriate benchmark for comparison with our real-world dataset. The PFS curves of the real-world ABVD patients and RATHL trial patients, aged younger than 60 years, are remarkably similar in both the absolute 5-year estimates of progression-free survival and in the kinetics of relapse. This is observed in both the whole ABVD cohort and IPS 3+ subgroup, and is consistent with data published from the Netherlands that suggest similar outcomes are achieved for HL patients who are treated within or outside of clinical trials [7].

It is clear from RATHL that higher-risk (IPS 3+) patients are more likely to be iPET2 positive after ABVD induction therapy, and despite treatment intensification with eBEACOPP or BEACOPP-14, they also had a higher relapse rate (32.5%) at 3 years [2]. Whilst, intuitively, it seems appropriate to intensify therapy in iPET2-positive patients, this has not been tested in a randomised trial and the magnitude of benefit from this strategy is unknown. Notably, only 2.6% of real-world ABVD patients had treatment intensification to eBEACOPP after induction ABVD, compared with 16.1% of RATHL patients, yet there was no observed difference in PFS between the real-world ABVD and RATHL patients in either the whole ABVD cohort or IPS 3+ subgroup. This suggests that either the magnitude of benefit for treatment intensification based on an iPET2 scan is limited or that there are unknown competing factors influencing the data.

In both our study and clinical trials, it seems that higher-risk patients achieve the greatest relative gain in PFS when treated with upfront eBEACOPP compared with ABVD. The RATHL trial has shown that even with interim-PET/CT-directed treatment intensification, IPS 3+ patients have an inferior PFS compared with IPS 0–2 patients. This is not solely explained by fewer patients achieving an iPET2-negative remission, as those IPS 3+ patients who achieved an iPET2-negative remission had a higher risk of relapse compared with IPS 0–2 patients (18.3% vs 13.1% at 3 years, HR 1.44 [1.04–2.01], *p*=0.029) [2] (Kirkwood, A, personal communication, 2016).

Although there has never been a randomised prospective trial that compared six cycles of eBEACOPP with six cycles of ABVD, randomised trials that have compared variations of these regimens have consistently shown a first-remission PFS advantage with upfront eBEACOPP, ranging from 5 to 18% [8–10]. The 5-year PFS difference between patients treated with upfront eBEACOPP compared with ABVD in our unselected real-world cohort was 16%. In keeping with prospective trial data, OS estimates were excellent in both of our treatment groups, suggesting that patients who relapse are frequently salvaged, albeit with the need for more toxic regimens. The comparative infrequency of relapse in real-world eBEACOPP patients correlates with their significantly lower likelihood of receiving an autologous stem cell transplant, compared with ABVD patients. Although not planned prospectively, there was clear clinician bias to treat IPS 3+ patients with upfront eBEACOPP in those centres where it was available. Notably, IPS 3+ eBEACOPP patients were also observed to have a disproportionate PFS benefit compared with IPS 3+ ABVD patients.

With regard to OS, IPS 3+ real-world eBEACOPP patients had an OS advantage compared with IPS 3+ real-world ABVD patients and showed a difference of just over 8% in 5-year OS when compared with IPS 3+ RATHL patients. Although this did not reach statistical significance, this may be due to the small number of patients and events, particularly within the real-world cohort (1 event in 33 patients). This difference is not that dissimilar to differences shown in meta-analyses comparing upfront ABVD to BEACOPP [11] which saw 5-year OS differences of 7% between ABVD (88% 5-year OS) and 6 cycles of eBEACOPP (95%).

As treatments for first line and relapsed Hodgkin lymphoma evolve and more options become available for our patients, the number of younger adults dying from Hodgkin lymphoma is thankfully decreasing. However, all treatment options at relapse bring more toxicities for patients, particularly when transplant strategies are employed, and achieving a durable first remission remains a highly important goal for our patients. With the publication of the HD18 and AHL2011 trials [12, 13], the cumulative toxicity of eBEACOPP strategies has been reduced with no impact on PFS, making the eBEACOPP strategy even more appealing as a first-line treatment option for advanced stage HL. Indeed, with longer follow-up of HD18, patients who achieved an interim metabolic remission after 2 cycles of eBEACOPP had an improved overall survival when treated with a total of 4 cycles of eBEACOPP compared with 6–8 cycles, owing to a reduction in treatment-related mortality [14]. This confirms that PET-guided strategies can optimise the PFS and OS for the majority of eBEACOPP-treated patients, whilst reducing the overall toxicity of the regimen.

Reduced fertility is a significant concern for patients treated with eBEACOPP. We reviewed the outcomes for menstrual cycle recovery and fertility after eBEACOPP and found consistent premature ovarian failure in pre-menopausal women aged over 30 years at diagnosis (*n*=4), with only one patient having a transient return of menstrual periods. Of the 13 pre-menopausal women treated with eBEACOPP aged under 30 years at diagnosis, the majority (*n*=11, 84.6%) regained menstrual periods and several pregnancies have been carried to term. Using anti-mullerian hormone levels as a surrogate for fertility, prospective follow-up of female patients from the RATHL study has confirmed that age over 35 and treatment with BEACOPP were both independent predictors of reduced AMH levels post-treatment, whilst the majority of woman under 35 treated with ABVD/AVD regained pre-treatment AMH levels by 2 years post-treatment [15]. Reducing the number of cycles of eBEACOPP is likely to help preserve fertility. Prospective trials that use interim-PET/CT assessment to de-escalate the better-risk patients after eBEACOPP induction therapy, such as in the recently published LYSA AHL2011, and HD18 trials, have shown encouraging results [12, 16]. It is now general practice in the UK to offer pre-treatment fertility counselling, including potential oocyte storage, to all women of reproductive age when clinically appropriate.

Second primary malignancies (SPM) are another concern for patients receiving first-line therapy for Hodgkin lymphoma and relative risks remain elevated for patients compared with age-matched controls whether treated with chemotherapy alone or in combination with radiotherapy. Meta-analysis of large trial datasets suggests an increased risk of therapy-related acute myeloid leukemia/myelodysplastic syndromes in patients treated with dose-intensified therapy [12]. However, as seen with our real-world dataset, patients treated with ABVD are more likely to need dose intensification after an interim PET scan or at relapse, so it remains difficult to advise patients of their individual long-term risk of a SPM when first-line therapy choices are made. With the RATHL patients to date, 4 non-cutaneous SPMs have been diagnosed in 154 patients who were dose escalated to BEACOPP-based chemotherapy (Kirkwood, A; personal communication).

In conclusion, our data provide further evidence that the outcomes for frontline treatment of young adults (aged younger than 60 years) with advanced-stage HL in a real-world setting are highly similar to those achieved in prospective clinical trials. Although RATHL-style intensification of therapy for iPET2-positive patients has become an accepted standard of care in the UK, 5-year PFS estimates for RATHL patients, aged 18–59 years, were highly similar to patients treated in a multicentre real-world setting with no iPET2-guided intensification strategy. We observed a clear PFS benefit for patients treated with upfront eBEACOPP and recent UK data have shown that since the publication of HD18, there has been a marked increase in the number of UK centres using eBEACOPP/eBEACOPDacarbazine as first-line therapy to treat advanced stage HL [17].

## Data Availability

Due to privacy and ethical concerns, the real-world data analysed in this study is not publicly available.
